# Dietary supplemental coated essential oils and organic acids mixture improves growth performance and gut health along with reduces *Salmonella* load of broiler chickens infected with *Salmonella* Enteritidis

**DOI:** 10.1186/s40104-023-00889-2

**Published:** 2023-07-01

**Authors:** Zeqiong Hu, Lin Liu, Fangshen Guo, Jia Huang, Jianing Qiao, Ruichen Bi, Jinyu Huang, Kaichen Zhang, Yuming Guo, Zhong Wang

**Affiliations:** 1 State Key Laboratory of Animal Nutrition, College of Animal Science and Technology, China Agricultural University, Beijing, China; 2 Shanghai Meinong Biotechnology Co., Ltd., Shanghai, China; 3 Shandong Heyi Food Co., Ltd., Zaozhuang City, Shandong Province China

**Keywords:** Broiler chickens, Essential oils and organic acids mixture, Gut health, *Salmonella* Enteritidis

## Abstract

**Background:**

Reducing *Salmonella* infection in broiler chickens by using effective and safe alternatives to antibiotics is vital to provide safer poultry meat and minimize the emergence of drug-resistant *Salmonella* and the spread of salmonellosis to humans. This study was to first evaluate the protective efficacy of feeding coated essential oils and organic acids mixture (EOA) on broiler chickens infected with *Salmonella* Enteritidis (*S*. Enteritidis, SE), and then its action mechanism was further explored.

**Methods:**

A total of 480 1-day-old Arbor Acres male chickens were randomly assigned into five treatments with six replicates, including non-challenged control fed with basal diet (A), SE-challenged control (B), and SE-infected birds fed a basal diet with 300 mg/kg of EOA (BL), 500 mg/kg of EOA (BM) and 800 mg/kg of EOA (BH), respectively. All birds on challenged groups were infected with *Salmonella* Enteritidis on d 13.

**Results:**

Feeding EOA showed a reversed ability on negative effects caused by SE infection, as evidenced by decreasing the feed conversion rate (FCR) and the ratio of villus height to crypt depth (VH/CD) (*P* < 0.05), obviously decreasing intestinal and internal organs *Salmonella* load along with increasing cecal butyric acid-producing bacteria abundance (*P* < 0.05). Moreover, supplemental different levels of EOA notably up-regulated claudin-1 (*CLDN-1*), occludin (*OCLN*), zonula occludens-1 (*ZO-1*), mucin-2 (*MUC-2*), fatty acid binding protein-2 (*FABP-2*), nuclear factor kappa-light-chain-enhancer of activated B cells (*NF-κB*), myeloid differential protein-88 (*MyD88*) and interleukin-6 (*IL-6*) mRNA levels in the ileum of the infected chickens after challenge, whereas down-regulated toll-like receptor-4 (*TLR-4*) mRNA levels (*P* < 0.05). Linear discriminant analysis combined effect size measurements analysis (LEfSe) showed that the relative abundance of *g_Butyricicoccus*, *g_Anaerotruncus* and *g_unclassified_f_Bacillaceae* significantly was enriched in infected birds given EOA. Also, phylogenetic investigation of communities by reconstruction of unobserved states (PICRUSt) analysis showed that alpha-linolenic acid metabolism, fatty acid metabolism and biosynthesis of unsaturated fatty acids were significantly enriched in the EOA group.

**Conclusion:**

Our data suggest that the essential oils and organic acids mixture can be used as an effective strategy to ameliorate and alleviate *Salmonella* Enteritidis infection in broilers.

## Introduction

*Salmonella enterica* serotype Enteritidis (*S.* Enteritidis, SE) is one of the remarkable foodborne pathogens that endanger the health of broiler chickens and poultry products safety. Infection of SE in chickens can destroy the balance of intestinal flora, adhere to intestinal epithelial cells, induce intestinal inflammation and damage intestinal barrier, resulting in diarrhea and growth loss of infected chickens [1–3]. In addition, *Salmonella* which breaks through the intestinal mucosal barrier and invades into the body can be colonized in internal organs such as spleen and liver, resulting in bacteremia and chicken death [4]. Traditionally, the addition of antibiotics to feed or water have been the main strategy of preventing and controlling salmonellosis in animal production [5]. However, many countries, including China, have gradually begun to ban the use of antibiotics in livestock production due to the continued emergence of antibiotic resistant strains and drug residues in poultry products [6]. Additionally, consumption of contaminated eggs or chicken meat is one of the leading causes of *Salmonella* food poisoning in humans [7]. Therefore, it is becoming more important to search effective and safe antibiotic substitute incorporation into feed and/or drinking water as a pre-harvest strategy to reduce *Salmonella* incidence and prevalence in poultry at the farm level.

In recent years, many relevant studies have reported that natural plant extracts such as essential oils, acidifiers, probiotics and their metabolites can effectively inhibit or kill *Salmonella*, improve the growth performance of livestock and poultry, reduce morbidity and mortality, and have the potential to become a substitute for antibiotics [6, 8, 9]. Poultry’s trials found that some essential oils, such as carvacrol, thymol, trans-cinnamaldehyde and eugenol have antibacterial effects against *Salmonella* in chickens [10–14], and could improve performance and reduce mortality and morbidity in broilers [15, 16]. Plant essential oils can exert their biological functions by affecting bacterial biofilms and destroying ion gradients [17], effectively scavenging nitric oxide [18], inhibiting the oxidation of low density lipoprotein and the expression of cyclooxygenase-2 and activating peroxisome proliferator activated receptors α and γ [19, 20]. Additionally, organic acids such as formic acids, butyric acid, medium chain fatty acid caprylic acid and benzoic acid have gained wide application in livestock production due to their possessing a variety of functions such as antibacterial (such as *Salmonella*, *Campylobacter, Clostridium perfringens* and other pathogens), immune-regulation, barrier-protection, health-promotion and/or growth promoters in chickens [8, 21–23].

Interestingly, previous studies have indicated that dietary essential oils combined with organic acids supplementation not only showed synergistic beneficial effects on growth performance and gut health, but also exhibited higher efficacy in controlling harmful intestinal bacterial infection such as *Escherichia coli, Salmonella* spp*.* and *Clostridium perfringens* [24–28], compared with individual addition. In addition, our previous studies have demonstrated that dietary supplementation with a blend product of essential oils and organic acids (4% carvacrol, 4% thyme, 0.5% hexanoic, 3.5% benzoic, and 0.5% butyric acid) could improve growth performance and intestinal health in broilers challenged with necrotic enteritis, and could be used as in-feed antibiotic alternative in broiler production [27, 28]. However, the efficacy of a blend of essential oils and organic acids for chicken growth performance and gut health was influenced by many factors, such as, the properties of essential oils (EOs) or organic acids (OAs), essential oils and organic acids (EOA) formula composition, protected EOA or not, EOA dosage, chicken health status, diet composition, and housing environment hygienic conditions [29]. A commercial blend product of coated essential oils and organic acids which contains thymol > 8.0%, carvacrol > 8.0%, cinnamaldehyde > 5%, caprylic acid > 1.0%, benzoic acid > 6.0%, butyric acid > 1.0% and carrier was used in the current study. In vitro studies have confirmed that the EOA product exhibited strong antibacterial activity against *Salmonella*, and the lowest minimum inhibitory concentration and minimum bactericidal concentration values against SE of this EOA was 2.35 and 4.69 mg/mL, respectively (unpublished data). The purpose of this study was to assess the effect of dietary inclusion of the EOA on growth performance and intestinal health of *Salmonella*-infected broilers chickens, and then action mechanism was explored.

## Materials and methods

### Animal ethics statement

All animal experiments were approved by the China Agricultural University Animal Care and Use Committee, Beijing, P. R. China (approval number: AW51112202-1–2).

### Experimental design and diets

Four hundred and eighty (*n* = 480) 1-day-old *Salmonella*-free male Arbor Acres (AA) broiler chickens were purchased from a local supplier (Beijing Arbor Acres Poultry Breeding Company, China). These birds were randomly divided into five treatments according to their initial body weight including: negative control group (A, neither EOA treatment nor SE infection), positive control group (B, SE infection but without EOA treatment) and infected birds given the basal diets with three levels of EOA-treated groups, respectively. Namely, BL, SE with 300 mg/kg EOA treatment; BM, SE with 500 mg/kg EOA treatment; and BH, SE with 800 mg/kg EOA treatment. Each treatment group had six replicates with 16 birds per replicate. Each replicate was housed in a separate cage (240 cm × 60 cm × 60 cm) to avoid direct physical contact of the birds and minimize cross-contamination among isolators. The un-medicated pelleted basal diet was formulated according to the American National Research Council (NRC) (1994) [30] broiler feeding standard. The composition and nutrient levels of the basal diet is shown in Table 1.

**Table 1:** Composition and nutrient levels of the basal diets

Items	d 1–21	d 22–42
Ingredient, %		
Corn (CP 8.0%)	51.30	53.25
Soybean meal (CP 44%)	37.00	33.50
Wheat powder (CP 13.5%)	4.20	5.00
Soybean oil	4.20	5.00
*DL*-Methionine, 99%	0.25	0.15
*L*-Lysine HCl, 78%	0.25	0.20
Limestone	1.12	1.00
Dicalcium phosphate	1.00	1.20
Sodium chloride	0.35	0.35
Choline chloride, 50%	0.12	0.15
Vitamin premix^a^	0.02	0.02
Mineral premix^b^	0.10	0.10
NSP enzyme^c^	0.02	0.02
Phytase	0.02	0.01
Antioxidant^d^	0.05	0.05
Total	100.00	100.00
Nutrient levels^e^		
Metabolizable energy, Mcal/kg	3.03	3.10
Crude protein, %	21.45	20.05
Total calcium, %	0.77	0.76
Total phosphorus, %	0.57	0.59
Available phosphorus, %	0.27	0.30
Lysine, %	1.33	1.21
Methionine, %	0.56	0.44
Methionine + Cystine, %	0.90	0.77
Threonine, %	0.80	0.75
Tryptophan, %	0.25	0.23

^a^Vitamin premix provided per kilogram of complete diet: vitamin A, 10,000 IU; vitamin D_3_, 2,400 IU; vitamin E, 20 IU; vitamin K_3_, 2.0 mg; vitamin B_1_, 1.6 mg; vitamin B_2_, 6.4 mg; vitamin B_6_, 2.4 mg; vitamin B_12_, 0.020 mg; nicotinic acid, 30 mg; pantothenic acid, 9.2 mg; folic acid, 1.0 mg; biotin, 0.10 mg

^b^Mineral premix provided per kilogram of complete diet: iron, 40 mg; copper, 8 mg; manganese, 60 mg; zinc, 55 mg; iodine, 0.75 mg; selenium, 0.15 mg

^c^NSP enzyme: non-starch polysaccharide enzyme

^d^Antioxidant: 33% ethoxyquinoline

^e^Calculated value based on the analyzed data of experimental diets

All chickens were kept in an environmentally controlled house and had free access to feed and water throughout the entire experimental period. In accordance with the AA  Broiler Management Guide, room temperature was maintained at 32 to 34 °C during d 1 to 5, and then gradually decreased by 2 °C weekly to reach a final room temperature of 22 to 24 °C. Artificial light was provided in a 23 h light/1 h dark program during the whole period of the study. In addition, the chickens were vaccinated against Newcastle disease virus and infectious bronchitis virus vaccines on d 7 and 21, and against infection bursa disease virus by drinking water on d 12 and 26, respectively.

### *Salmonella* Enteritidis culture and challenge protocol

*Salmonella* Enteritidis serotype CVCC3379 (China Veterinary Culture Collection Center, China Institute of Veterinary Drug Control, Beijing, China) was cultured in nutrient broth (CM106, NB, Beijing Land Bridge Technology Co., Ltd., Beijing, China) at 37 °C with orbital shaking for 16 h. The concentration of viable SE in the culture was counted on *Salmonella Shigella* agar (CM206, SS, Beijing Land Bridge Technology Co., Ltd., Beijing, China) at 37 °C for 24 h and the stock culture was adjusted to a final concentration of 1 × 10^9^ CFU/mL SE. On d 13, birds in the SE-challenged groups were administered 1.0 mL of bacterial suspension containing approximately 1 × 10^9^ CFU/mL of SE suspension by gavage. Unchallenged groups received 1.0 mL of phosphate buffered saline (PBS) without SE on the same date. Feed was withdrawn from all birds for 10 h before challenge.

### Growth performance

Dead birds were recorded daily and the mortality rate of each replicate was calculated through the experiment. Body weight (BW) and feed of the birds were weighed on a per cage basis on d 0, 23 and 39. Average body weight (ABW), average daily gain (ADG), average daily feed intake (ADFI), and feed conversion ratios (FCR) were calculated and corrected for mortality rate for each feeding stage at different experimental period.

### Sample collection

On 3 days post infection (DPI) and 10 DPI, one bird from each replicate pen was randomly selected, weighed, blood samples were collected from the wing vein and centrifuged (3,000 × *g*, 10 min) at 4 °C, and then the serum was harvested and stored at –20 °C until analysis. The birds were euthanized by cervical dislocation. The middle intestinal sections of the ileum were cut out (approximately 200 mg), gently washed with ice-cold sterile saline, then put into a sterile tube and immediately snap-frozen in liquid nitrogen solution and stored at –80 °C for mRNA expression determination. Another ileal sample (approximately 1 cm) was rinsed in 0.9% (w/vol) physiological saline and fixed in 4% (w/vol) paraformaldehyde buffer solution for later morphological analysis. Liver and spleen samples (approximately 2 g, respectively) from each killed bird were aseptically collected into sterile tubes, then immediately snap-frozen in liquid nitrogen, stored at –40 °C for the determination of *Salmonella* translocation. The cecal contents of each killed bird were aseptically collected, put into three sterile tubes, then immediately snap-frozen in liquid nitrogen and transferred to –80 °C for microbial culture, microbial 16S rRNA analysis and the measurement of short-chain fatty acids (SCFA) contents. Ileal mucosa was collected and homogenized in ice-cold PBS (pH 7.2), and centrifuged, then the supernatant was collected and stored at –20 °C for anti-*Salmonella* specific IgA determination.

### Determination of bacteria in cecal contents and internal organs

*Salmonella* enumeration in the cecal contents and internal organ were determined as described previously [31]. Briefly, liver, spleen and cecal samples were weighed, tenfold diluted with sterile saline (w/v) and homogenized for 1 min using a stomacher respectively. The homogenate was further serially diluted tenfold (1:10) with sterile PBS to appropriate levels, then 100 μL of each dilution was plated onto selective ager plates for bacterial quantification, respectively. *Salmonella* and *Escherichia coli* were counted with *Salmonella Shigella* agar (CM206, SS, Beijing Land Bridge Technology Co., Ltd., China) and Eosin-Methylene Blue Agar (CM105, EMB, Beijing Land Bridge Technology Ltd., China) by aerobical incubation at 37 °C for 24 h respectively. *Lactobacillus* spp. were determined with Man Rogosa Sharpe Medium (HB0384, MRS, Qingdao HopeBio Technology Co., Ltd., Shandong Province, China) by anaerobical culture for 24–48 h at 37 °C. *Campylobacter* were incubated by using modified Charcoal Cefoperazone Deoxycholate agar (HB0274, mCCDA, Qingdao HopeBio Technology Co., Ltd., Shandong Province, China) supplemented with CCDA selective supplement, and incubated microaerobically at 42 °C for 48–96 h using Anaero Jars (AG0025A, Thermo Fisher Scientific, Waltham, MA, United States). The number of colony-forming units in spleen, liver and cecal digesta was expressed as a logarithmic transformation per gram. Subsequently, the liver and spleen samples of all unchallenged chickens were enriched in tetrathionate broth base (HB4086, TTB, Qingdao HopeBio Technology Co., Ltd., Shandong Province, China) and further incubated at 37 °C for 24 h. Enrichment samples were confirmed negative for *Salmonella* spp. by streak plating on *Salmonella Shigella* agar selective media.

### Ileum morphology analysis

Gut morphology analysis was performed as previously described [32]. The fixed tissue samples were dehydrated in a tissue processor (Leica Microsystems K. K., Tokyo, Japan), and embedded in paraffin wax. Paraffin sections. (5 μm) were sliced serially using a microtome (Leica Microsystems K. K., Tokyo, Japan) and mounted on glass slides. The paraffin was removed by xylene (2 times for 5 min each), followed by rehydration in 95% alcohol (5 min) and 50% alcohol (5 min). Sections were stained with haematoxylin and eosin (HE) for villous morphology measurement. The villi height (VH) and crypt depth (CD) of the stained sections were measured using image processing and analyzing system (at 40 × combined magnification, Inverted microscope: NIKON CI-S, Tokyo, Japan; Imaging system: NIKON DS-U3, Tokyo, Japan; CaseViewer 2.3, JAVS, Inc.). Ten intact villi were selected for measurement.

### Determination of gene expression in the ileum using quantitative real-time polymerase chain reaction (RT-PCR)

Extraction of total RNA in ileum (50–100 mg) was performed by using Trizol reagent (Tiangen Biotech Co., Ltd., Beijing, China) according to the manufacturer’s instructions. The purity and concentration of total RNA were measured using a NanoDrop-2000 spectrophotometer (Thermo Fisher Scientific, Waltham, MA, United States). Then, cDNA was synthesized by using PrimeScript™ RT reagent Kit with gDNA Eraser (Perfect Real Time) kit (Takara BioTechnology Co., Ltd., Beijing, China). Quantitative real-time PCR (qRT-PCR) reactions were performed in the Applied Biosystems’ 7500 Fast Real-Time PCR system by using SYBR Premix Ex Taq diagnostic kit (Takara BioTechnology Co., Ltd., Beijing, China) and each sample was measured in duplicate. *β-actin* gene was used as housekeeping control to normalize variations in the mRNA amount for the target genes including *OCLN*, *ZO-1*, *MUC-2*, *CLDN-1*, *FABP-2*, *NF-κB*, *TLR-4*, *MyD88*, *IL-6*, interleukin-1β (*IL-1β*), tumor necrosis factor-α (*TNF-α*) and interferon-γ (*IFN-γ*). The sequences of gene primers used in this study are shown in Table 2. Relative target gene expression level of each target gene was normalized by the comparative cycle threshold (CT) 2^−ΔΔCT^ method [33].

**Table 2:** Sequences of the oligonucleotide primers used for quantitative real-time PCR^a^

Gene	Primer sequence (5′→3′)	GenBank ID
Barrier-related genes		
*CLDN-1*	F: CATACTCCTGGGTCTGGTTGGT	NM_001013611.2
	R: GACAGCCATCCGCATCTTCT	
*OCLN*	F: ACGGCAGCACCTACCTCAA	NM_205128.1
	R: GGGCGAAGAAGCAGATGAG	
*ZO-1*	F: CTTCAGGTGTTTCTCTTCCTCCTC	XM_040706827.1
	R: CTGTGGTTTCATGGCTGGATC	
*MUC-2*	F: TTCATGATGCCTGCTCTTGTG	XM_421035
	R: CCTGAGCCTTGGTACATTCTTGT	
*FABP-2*	F: GAAGCAATGGGCGTGAATGTGATG	NM_001007923.1
	R: TTCGATGTCGATGGTACGGAAGTTG	
Immune-related genes		
*NF-κB*	F: TGGAGAAGGCTATGCAGCTT	NM_205134.1
	R: CATCCTGGACAGCAGTGAGA	
*TLR-4*	F: CCACTATTCGGTTGGTGGAC	NM_001030693.1
	R: ACAGCTTCTCAGCAGGCAAT	
MyD88	F: TGCAAGACCA TGAAGAACGA	NM_001030962.3
	R: TCACGGCAGCAAGAGAGATT	
*IL-6*	F: GATCCGGCAGATGGTGATAA	NM_204628.1
	R: AGGATGAGGTGCATGGTGAT	
*IL-1β*	F: TCATCTTCTACCGCCTGGAC	NM_204524.1
	R: GTAGGTGGCGATGTTGACCT	
*TNF-α*	F: GAGCGTTGACTTGGCTGTC	NM_204267.1
	R: AAGCAACAACCAGCTATGCAC	
*IFN-γ*	F: CTTCCTGATGGCGTGAAGA	NM_205149.1
	R: GAGGATCCACCAGCTTCTGT	
*β-actin*	R: GAGAAATTGTGCGTGACATCA	NM_205518.1
	F: CCTGAACCTCTCATTGCCA	

^a^Primers were designed and synthesized by Sangon Biotech (Shanghai) Co., Ltd. (China). *F* Forward, *R* Reverse

*CLDN-1* Claudin-1, *FABP-2* Fatty acid binding protein, *IFN-γ* Interferon-γ, *IL-1β* Interleukin-1β, *IL-6* Interleukin-6, *MUC-2* Mucin-2, *MyD88* Myeloid differential protein-88, *NF-κB* Nuclear factor kappa-light-chain-enhancer of activated B cells, *OCLN* Occludin, *TLR-4* Toll-like receptor-4, *TNF-α* Tumor necrosis factor-α, *ZO-1* Zonula occludens-1

### Measurement of anti-*Salmonella* specific antibody in the serum and ileal content

Serum anti–SE specific immunoglobulin G (IgG) and specific immunoglobulin A (IgA) in ileal content were measured using an indirect enzyme-linked immunosorbent assay (ELISA) as described previously [32]. Briefly, SE (1 × 10^8^ CFU/mL) cells were washed 3 times with sterile PBS (pH 7.2) and lysed by an ultrasonic processor JY96-IIN (Ningbo Xinzhi Biotechnology Co., Ltd., China) at 85 Watts and 30 s intervals on ice for 5 min. The lysed cells were centrifuged at 10,000 × *g* for 10 min, and the resultant supernatant was collected and stored at –70 °C until use. Protein concentration of the lytic supernatant of *Salmonella* bacteria was determined by bicinchoninic acid kit (G2026-200 T, Wuhan ServiceBio Technology, Co., Ltd., China). Flat-bottomed 96-well ELISA microtiter plates (Corning Costar, Corning, NY, USA) were incubated with 100 μL/well of the prepared *Salmonella* lytic supernatant (20 μg/mL) dissolved in 0.1 mol/L carbonate-bicarbonate buffer (15 mmol/L Na_2_CO_3_, 35 mmol/L NaHCO_3_, 0.3 mmol/L NaN_3_) overnight at 4 °C. Antigen-coated plate was then washed 3 times with PBST (phosphate buffered saline pH 7.2 containing 0.05% Tween X-100), 200 μL of blocking solution (PBST containing 1% bovine serum albumin) was added to each well and incubated at 37 °C for 2 h for blocking nonspecific binding. After washing 3 times with PBST, 100 μL of diluted serum samples or intestinal mucosa supernatant were added to each well, respectively, and incubated for 1 h at 37 °C. After washing, 100 μL of diluted horseradish peroxidase (HRP)-conjugated goat anti-chicken IgG (A30-104P, Bethyl Laboratories Inc., Montgomery, TX) or HRP- conjugated goat anti-chicken IgA-Fc (A30-103P, Bethyl Laboratories Inc., Montgomery, TX) were added to each well, and incubated at 37 °C for 1 h. The plates were washed 3 times with PBST and incubated with 3,3′,5,5′-tetra-methylbenzidine solution for 30 min at room temperature in the dark. Finally, the reaction was stopped with 2 mol/L sulfuric acid, and the absorbance was measured at 450 nm using an automatic ELISA reader (Bio-Tek EL311SX autoreader, Bio-Tek, USA). The result is presented as an optical density (OD) value.

### Determination of short chain fatty acids concentration in cecal content

A total of 100 mg of frozen cecal digesta sample of each replicate was dissolved and homogenized in 1.5 mL of pre-cold sterile ultra-pure water, and then centrifuged (12,000 × *g*, 10 min at 4 °C). Then, 1 mL of the supernatant was diluted with 0.2 mL of 25% (w/v) metaphosphoric acid solution containing crotonic acid. The mixture was incubated at –20 °C for 24 h and then centrifuged (10,000 × *g*, 10 min at 4 °C) to remove protein precipitates. The extracted solution was filtered with a 0.22-μm syringe filter, and then analyzed short chain fatty acids (SCFAs) using a gas chromatograph (Shimadzu GC-2014 ATF instrument) equipped with a capillary column (30 m × 0.25 mm × 0.5 μm). The N_2_ was used for carrier gas (12.5 Mpa, 18 mL/min). The temperature of the injector and detector was 180 °C, and the column was gradually heated from 80 °C to 170 °C at a rate of 5 °C/min. The results of SCFAs were expressed as milligrams per kilogram of digesta.

### Microbial DNA extraction, 16S rRNA gene amplification, sequencing and bioinformatics analysis

Microbial genomic DNA was extracted from about 250 mg cecal digesta samples taken from all groups, respectively, using E.Z.N.A.® Soil DNA Kit (Omega Bio-Tek, GA, USA) according to the manufacturers’ instructions. The concentration and purity of total DNA were detected by NanoDrop 2000 spectrophotometer (Thermo Scientific, MA, USA), and the integrity of DNA was detected by 1% agarose gel electrophoresis (voltage 5 V/cm, time 20 min). The V3–V4 regions of bacterial 16S rDNA sequences were amplified using primer 338F (5′-ACTCCTACGGGAGGCAGCAG-3′) and 806R (5′-GGACTACHVGGGTWTCTAAT-3′) according to the method described previously [28]. The PCR product was purified using AxyPrep DNA Gel Extraction Kit (Axygen, Union City, USA), quantified, homogenized, and then constructed the Miseq library. The library was sequenced by the Illlumina MiSeq PE250 platform (Illumina, Santa Clara, CA, USA) using a MiSeq Reagent Kit at Shanghai Personal Biotechnology Co., Ltd. (Shanghai, China).

Raw pair-end sequences were demultiplexed and quality-filtered using Quantitative Insights Into Microbial Ecology (QIIME, version 1.17) [34]. The effective reads were clustered into operational taxonomic units (OTUs) based on the 97% similarity. Classification of OTUs at various taxonomic levels was carried out using the Greengenes database. For rarefaction curves and α-diversity (Chao 1 index, Simpson index, ACE index, Shannon index) analysis were calculated using QIIME software [35]. β-diversity was estimated using principal coordinate analysis (PCoA) and partial least squares discriminant analysis. The results were plotted using “vegan” and “ggplot2” package in R software (Version 3.4.4). The significance of microbial community differences among groups was assessed using ANOSIM with R package “vegan” [36]. Linear discriminant analysis (LDA) combined effect size (LEfSe) analysis (LDA score > 2.0, *P* < 0.05) estimated the impact of the abundance of bacteria on the difference effect of bacteria from phylum to genus among different groups. Non-parametric factorial Kruskal–Wallis sum-rank test was employed to explore the differences in the relative abundances of bacteria among groups [37]. Phylogenetic Investigation of Communities by Reconstruction of Unobserved States (PICRUSt 1.0.0) was used to predict metagenome functions associated with bacterial communities based on high-quality 16S rRNA sequencing data [38]. The functions were deduced using Kyoto Encyclopedia of Genes and Genomes annotations for level 3 pathways. Differentially represented functional pathways were analyzed with two-sided Welch’s *t*-test. The obtained biome file was processed by STAMP (Halifax, Nova Scotia, Canada) version 2.1.3 [39].

### Statistical analysis

Linear and quadratic relationship analysis and one-way analysis of variance (one-way ANOVA) followed by Duncan’s multiple comparison test (SPSS, version 21.0, Chicago, IL, USA) was employed to analyze the difference in growth performance, intestinal morphology, bacterial population, gene expression, specific antibody levels and SCFAs content. *P* < 0.05 was considered significant, while 0.05 ≤ *P* < 0.10 was considered a trend. Data were expressed as mean and pooled standard error of mean (SEM). Correlations were analyzed using spearman correlation with the p-heatmap package (*P* < 0.05).

## Results

### Growth performance

The growth performance results are summarized in Table 3. From d 1 to 23, SE-infected control group had the lowest ADG and the highest FCR compared with the other four groups, but there was no statistical difference (*P* > 0.05). From d 24 to 39, ADFI and FCR of BH group were significantly lower than those of other groups (*P* < 0.05), and both indexes exhibited a quadratic change with increasing levels of EOA (*P* < 0.05). Moreover, the FCR of B and BL group was the highest among the five groups. During the overall period, although there were no significant differences in ABW, ADG and MOT among all groups from d 1 to 39 (*P* > 0.05), ADFI and FCR exhibited a quadratic change with increasing levels of EOA (*P* < 0.05). In addition, SE-induced increase in FCR was significantly inhibited by the addition of EOA into broiler diets compared to that in SE-infected control group.

**Table 3:** Effects of dietary EOA supplementation on growth performances of broiler chickens infected with *Salmonella* Enteritidis (*n* = 6)

Time	Items	Groups					SEM^1^	*P*-value		
		**A**	**B**	**BL**	**BM**	**BH**		***P1***^**2**^	**Linear**^**3**^	**Quadratic**^**4**^
d 1–23	ADG, g/bird/d	45.68	44.34	45.82	45.93	45.62	0.325	0.544	0.120	0.490
	ADFI, g/bird/d	61.06	60.22	60.78	62.91	61.55	0.479	0.524	0.132	0.027
	FCR^5^	1.34	1.38	1.32	1.36	1.34	0.011	0.371	0.120	0.985
d 24–39	ADG, g/bird/d	88.55	86.23	82.04	83.80	81.35	1.349	0.420	0.349	0.607
	ADFI, g/bird/d	143.34^ab^	144.58^b^	137.08^b^	138.47^ab^	130.79^c^	1.285	0.001	0.023	0.009
	FCR	1.61^b^	1.67^a^	1.67^a^	1.65^ab^	1.61^b^	0.009	0.021	0.582	0.008
d 39	ABW, g/bird	2,512.00	2,448.33	2,410.05	2,440.78	2,394.75	21.358	0.471	0.859	0.993
d 1–39	ADG, g/bird/d	63.29	61.67	60.67	61.45	60.29	0.732	0.398	0.679	0.775
	ADFI, g/bird/d	94.49^a^	94.87^a^	92.89^ab^	93.66^ab^	89.15^b^	0.747	0.090	0.388	0.027
	FCR	1.49^b^	1.54^a^	1.53^a^	1.52^a^	1.48^b^	0.008	0.015	0.817	0.020
	MOT, %	1.62	1.39	2.58	1.25	1.18	0.638	0.891	0.612	0.543

^1^*SEM* Standard error of the mean

^2^*P1*-value represent the difference comparison between group A, B, BL, BM and BH groups

^3^Linear regression analysis among B, BL, BM and BH groups

^4^Quadratic curve analysis among B, BL, BM and BH groups

^5^FCR = feed conversion ratio = g of feed intake/g of body weight gain, g/g

^a–c^Means within the same row without a common superscript differ significantly (*P* < 0.05)

*ADG* Average daily gain, *ADFI* Average daily feed intake, *ABW* Average body weight, *MOT* Mortality. A: broiler chickens with basal diet and no infection, B: broiler chickens with basal diet and SE infection, BL: broiler chickens with basal diet supplemented with 300 mg/kg EOA and SE infection, BM: broiler chickens with basal diet supplemented with 500 mg/kg EOA and SE infection, BH: broiler chickens with basal diet supplemented with 800 mg/kg EOA and SE infection. *EOA* Coated essential oils and organic acids mixture

### Ileal morphology

**Fig. 1:**
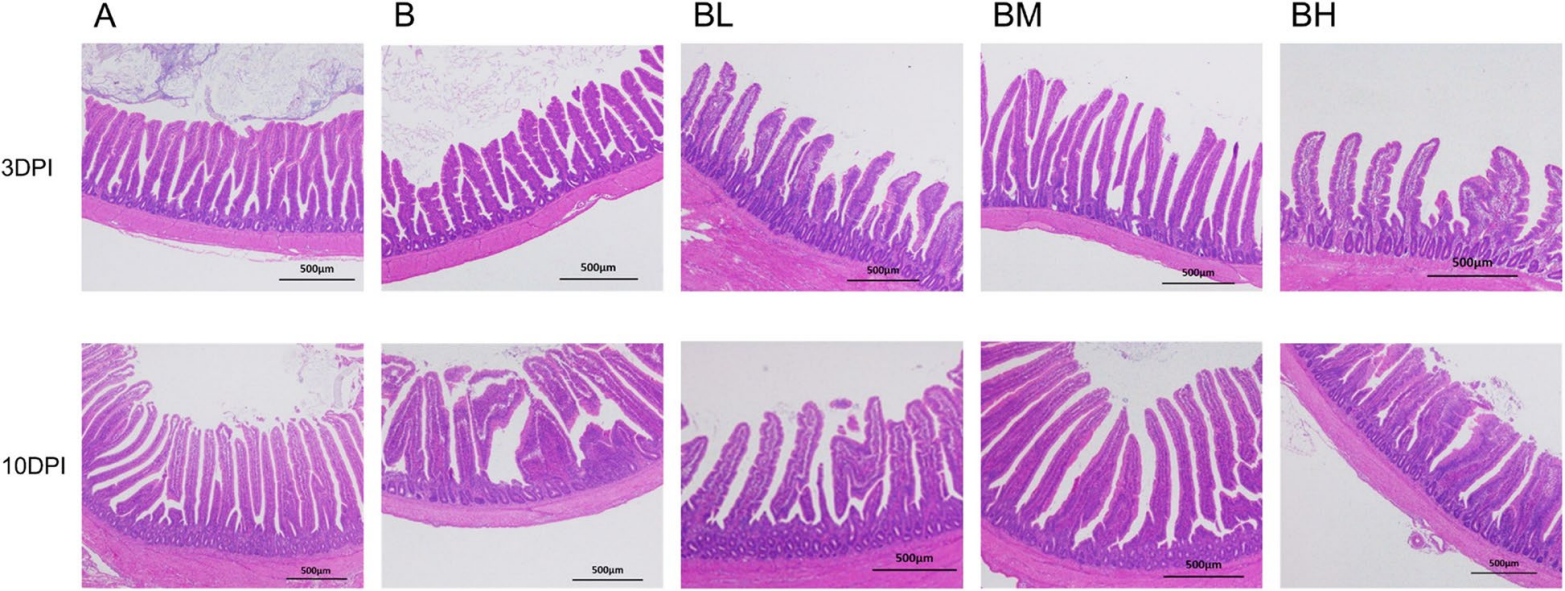
Effects of dietary EOA supplementation on gut morphological structure (× 40 magnification; scale bar: 500 μm) of the SE-infected broiler chickens. A: broiler chickens with basal diet and no infection, B: broiler chickens with basal diet and SE infection, BM: broiler chickens with basal diet supplemented with 500 mg/kg EOA and SE infection, BH: broiler chickens with basal diet supplemented with 800 mg/kg EOA and SE infection. DPI: days post infection. EOA: coated essential oils and organic acids mixture. SE: *Salmonella* Enteritidis

Figure 1 shows that the height of ileal villi was short and there was severe rupture of intestinal villi in the positive group. The addition of EOA can prevent ileal injury and improve the condition of ileal villi to some extent. As shown in Table 4, the VH/CD values in SE-infected B group was significantly lower than that in negative group and BM group at 3 DPI (*P* < 0.05). At 10 DPI, the CD of ileum in B group was significantly higher than that in the other four groups (*P* < 0.05), while the VH/CD in group B was significantly lower than that in negative group and BM group (*P* < 0.05). What's more, CD and VH/CD showed a linear change with increasing levels of EOA supplementation (*P* < 0.05).

**Table 4:** Effects of dietary EOA supplementation on ileal morphology of broiler chickens infected with *Salmonella* Enteritidis (*n* = 6)

Items	Groups					SEM^1^	*P*-value		
	**A**	**B**	**BL**	**BM**	**BH**		***P1***^**2**^	**Linear**^**3**^	**Quadratic**^**4**^
3 DPI									
Villus height, μm	621.18	539.93	594.39	590.57	580.15	10.236	0.107	0.067	0.209
Crypt depth, μm	101.08	105.56	105.10	95.26	100.29	1.919	0.477	0.183	0.920
VH/CD	6.20^a^	5.15^b^	5.70^ab^	6.21^a^	5.79^ab^	0.132	0.040	0.012	0.326
10 DPI									
Villus height, μm	699.76	695.11	672.05	761.26	696.20	19.022	0.714	0.650	0.982
Crypt depth, μm	104.75^b^	139.75^a^	109.94^b^	111.91^b^	109.27^b^	3.435	0.001	0.001	0.162
VH/CD	6.73^a^	5.11^b^	6.11^ab^	6.77^a^	6.36^ab^	0.209	0.043	0.003	0.364

^1^*SEM* Standard error of the mean

^2^*P1*-value represent the difference comparison between group A, B, BL, BM and BH groups

^3^Linear regression analysis among B, BL, BM and BH groups

^4^Quadratic curve analysis among B, BL, BM and BH groups

^a,b^Means within the same row without a common superscript differ significantly (*P* < 0.05)

DPI: days post infection, VH/CD: villus height to crypt depth ratio. A: broiler chickens with basal diet and no infection, B: broiler chickens with basal diet and SE infection, BL: broiler chickens with basal diet supplemented with 300 mg/kg EOA and SE infection, BM: broiler chickens with basal diet supplemented with 500 mg/kg EOA and SE infection, BH: broiler chickens with basal diet supplemented with 800 mg/kg EOA and SE infection. EOA: coated essential oils and organic acids mixture

### Caecal bacterial colonization and internal organs bacteria invasion

The results of plate count method showed that *Salmonella* and *Escherichia coli* were not detected in liver and spleen in negative group at 3 and 10 DPI. At 3 DPI, *Salmonella* was detected only in the liver (0.58 lgCFU/g) and spleen (0.33 lgCFU/g) of SE-infected control group and in the liver (0.40 lgCFU/g) in the BM group. Besides, *Escherichia coli* was detected in the livers of the four groups and the concentration of *Escherichia coli* in SE-infected control group was the highest (2.04 lgCFU/g) at 3 DPI. Notably, no *Salmonella* was detected in the liver and spleen of the four groups at 10 DPI. Similarly, the content of *Escherichia coli* was the highest in the spleen of SE-infected control group (1.17 lgCFU/g) at 10 DPI.

As summarized in Table 5, the numbers of *Salmonella* and *Lactobacillus* in the infected positive group were significantly higher than that in other groups at 3 DPI (*P* < 0.05). Dietary supplementation of EOA exhibited a significant linear decrease in the number of *Salmonella* and *Lactobacillus* in cecal digesta at 3 DPI (*P* < 0.05). At 10 DPI, *Salmonella* and *Campylobacter* counts in positive group were significantly higher than those in negative group (*P* < 0.05). Moreover, the number of *Salmonella, Escherichia coli* and *Campylobacter* in BL, BM and BH groups was lower than that in the positive group at 3 and 10 DPI. Therefore, the addition of EOA could suppress the increase of harmful bacteria in cecum of broilers caused by *Salmonella* challenge to some extent.

**Table 5:** Effects of EOA on microbial concentration (lgCFU/g)^1^ in the cecum contents of broilers infected with *Salmonella* Enteritidis (*n* = 6)

Items	Time	Groups					SEM^2^	*P*-value		
		**A**	**B**	**BL**	**BM**	**BH**		***P1***^**3,4**^	**Linear**^**5**^	**Quadratic**^**6**^
*Salmonella*	3 DPI	0.00	5.30^a^	4.00^b^	4.24^b^	3.82^b^	0.181	0.018	0.004	0.310
	10 DPI	0.00	5.46	5.06	4.75	4.53	0.203	0.481	0.140	0.770
*Escherichia coli*	3 DPI	4.70	5.62	4.98	4.75	4.72	0.138	0.168	0.024	0.734
	10 DPI	6.31	6.44	5.85	5.97	5.80	0.123	0.360	0.111	0.643
*Lactobacillus*	3 DPI	10.38^b^	11.28^a^	10.28^b^	9.78^b^	10.10^b^	0.139	0.001	0.001	0.207
	10 DPI	9.33^c^	11.02^a^	9.64^bc^	10.29^ab^	10.55^ab^	0.177	0.002	0.097	0.025
*Campylobacter*	3 DPI	5.61	6.45	6.12	6.17	6.07	0.106	0.142	0.164	0.796
	10 DPI	5.35^b^	6.76^a^	6.62^a^	6.25^a^	6.29^a^	0.146	0.006	0.138	0.779

^1^*lgCFU/g* log_10_ colony-forming units per gram of cecal digesta

^2^*SEM* Standard error of the mean

^3^*P*-value between B, BL, BM and BB groups in *Salmonella* content

^4^*P*-value represent the difference of other bacteria content among A, B, BL, BM and BB groups

^5^Linear regression analysis among B, BL, BM and BH groups

^6^Quadratic curve analysis among B, BL, BM and BH groups

^a–c^Means within the same row without a common superscript differ significantly (*P* < 0.05)

DPI: days post infection, A: broiler chickens with basal diet and no infection, B: broiler chickens with basal diet and SE infection, BL: broiler chickens with basal diet supplemented with 300 mg/kg EOA and SE infection, BM: broiler chickens with basal diet supplemented with 500 mg/kg EOA and SE infection, BH: broiler chickens with basal diet supplemented with 800 mg/kg EOA and SE infection. EOA: coated essential oils and organic acids mixture

### Gene expression of tight junction protein genes and immune-related genes in the ileum

Table 6 presents the results of ileal barrier-related gene expression in broilers. At 3 DPI, the mRNA levels of *CLDN-1*, *OCLN*, *ZO-1* and *MUC-2* in B, BL, BM and BH groups were significantly lower than those in non-infected A group (*P* < 0.05), indicating that SE infection damage intestinal barrier function. At 10 DPI, the gene expression of *CLDN-1*, *OCLN* and *MUC-2* in negative group was significantly higher than those in SE-infected B group (*P* < 0.05). Furthermore, our data showed that the gene expression of *CLDN-1*, *OCLN*, *ZO-1, MUC-2* and *FABP-2* in the ileum of BL, BM and BH groups was significantly higher than those in SE-infected B group (*P* < 0.05) and exhibited a quadratic change with increasing levels of EOA (*P* < 0.05). These data suggest that dietary supplementation of EOA can improve the expression of tight junction protein in the ileum of broilers challenged by SE.

**Table 6:** Effect of dietary EOA on mRNA expression of ileal tight junction proteins of broiler chickens infected with *Salmonella* Enteritidis (*n* = 6)

Items	Groups					SEM^1^	*P*-value		
	**A**	**B**	**BL**	**BM**	**BH**		***P1***^**2**^	**Linear**^**3**^	**Quadratic**^**4**^
3 DPI									
*CLDN-1*	2.72^a^	1.00^b^	1.35^b^	1.17^b^	1.03^b^	0.157	0.001	0.679	0.255
*OCLN*	1.83^a^	1.00^b^	1.05^b^	1.07^b^	0.60^b^	0.109	0.002	0.372	0.100
*ZO-1*	3.18^a^	1.00^b^	1.41^b^	1.30^b^	1.01^b^	0.193	0.001	0.356	0.025
*MUC-2*	1.83^a^	1.00^b^	1.22^b^	1.31^b^	1.19^b^	0.088	0.022	0.180	0.571
*FABP-2*	1.46	1.00	1.26	1.17	1.44	0.090	0.481	0.219	0.743
10 DPI									
*CLDN-1*	2.25^bc^	1.00^d^	2.46^b^	3.49^a^	1.68^c^	0.207	0.001	< 0.001	< 0.001
*OCLN*	1.80^b^	1.00^c^	2.78^a^	2.98^a^	1.32^bc^	0.188	< 0.001	0.002	< 0.001
*ZO-1*	1.32^bc^	1.00^c^	1.84^a^	1.70^ab^	1.66^ab^	0.083	0.002	0.001	0.033
*MUC-2*	3.17^b^	1.00^d^	4.99^a^	5.69^a^	2.07^c^	0.393	< 0.001	0.002	< 0.001
*FABP-2*	1.61^dc^	1.00^d^	5.68^a^	4.04^b^	1.93^c^	0.417	< 0.001	< 0.001	< 0.001

^1^*SEM* Standard error of the mean

^2^*P1*-value represent the difference comparison between group A, B, BL, BM and BH groups

^3^Linear regression analysis among B, BL, BM and BH groups

^4^Quadratic curve analysis among B, BL, BM and BH groups

^a–d^Means within the same row without a common superscript differ significantly (*P* < 0.05)

DPI: days post infection, A: broiler chickens with basal diet and no infection, B: broiler chickens with basal diet and SE infection, BL: broiler chickens with basal diet supplemented with 300 mg/kg EOA and SE infection, BM: broiler chickens with basal diet supplemented with 500 mg/kg EOA and SE infection, BH: broiler chickens with basal diet supplemented with 800 mg/kg EOA and SE infection. EOA: coated essential oils and organic acids mixture

The results of immune-related gene expression were listed in Table 7. The mRNA levels of *NF-κB*, *IL-1β* and *TNF-a* in BM group were significantly lower than those in SE-infected B group at 3 DPI (*P* < 0.05). The expression of inflammatory genes (*TLR4*, *MyD88*, *IL-6* and *IFN-γ*) in the four groups was also lower than that in SE-infected B group, but the difference was not significant. At 10 DPI, dietary supplementation of EOA showed a significant linear decreasing effect on *TLR4* mRNA level, displayed a quadratic effect on *NF-κB* and *MyD88* mRNA levels and had a significant linear and quadratic influence on *IL-6* mRNA levels (*P* < 0.05). Moreover, dietary different dosage of EOA administration all significantly reduced *TLR4* mRNA levels in the ileum (*P* = 0.002).

**Table 7:** Effect of dietary EOA on mRNA expression of ileal inflammatory genes of broiler chickens infected with *Salmonella* Enteritidis (*n* = 6)

Items	Groups					SEM^1^	*P*-value		
	**A**	**B**	**BL**	**BM**	**BH**		***P1***^**2**^	**Linear**^**3**^	**Quadratic**^**4**^
3 DPI									
*TLR4*	0.76	1.00	0.66	0.65	0.74	0.062	0.418	0.086	0.363
*NF-κB*	0.68^ab^	1.00^a^	0.58^ab^	0.51^b^	0.76^ab^	0.064	0.129	0.066	0.139
*MyD88*	1.08	1.00	0.96	0.81	0.89	0.063	0.711	0.509	0.973
*IL-6*	0.63	1.00	0.50	0.33	0.64	0.115	0.462	0.167	0.418
*IL-1β*	0.69^ab^	1.00^a^	0.56^ab^	0.42^b^	0.74^ab^	0.069	0.078	0.046	0.137
*TNF-a*	0.66^ab^	1.00^a^	0.94^a^	0.55^b^	0.92^a^	0.059	0.043	0.183	0.621
*IFN-γ*	0.88	1.00	0.50	0.33	0.64	0.094	0.281	0.597	0.822
10 DPI									
*TLR4*	0.89^ab^	1.00^a^	0.48^c^	0.66^bc^	0.36^c^	0.066	0.002	0.001	0.559
*NF-κB*	0.94^b^	1.00^b^	1.47^a^	1.20^ab^	0.96^b^	0.058	0.027	0.941	0.005
*MyD88*	1.35^b^	1.00^b^	2.69^a^	2.89^a^	1.35^b^	0.206	0.001	0.091	0.001
*IL-6*	0.86^c^	1.00^c^	2.59^a^	2.32^b^	1.70^b^	0.175	< 0.001	0.003	0.002
*IL-1β*	0.72	1.00	1.07	1.10	0.92	0.071	0.413	0.871	0.470
*TNF-a*	0.95	1.00	0.94	0.94	0.50	0.071	0.189	0.108	0.136
*IFN-γ*	0.55	1.00	0.94	1.06	1.15	0.101	0.330	0.711	0.648

^1^*SEM* Standard error of the mean

^2^*P1*-value represent the difference comparison between group A, B, BL, BM and BH groups

^3^Linear regression analysis among B, BL, BM and BH groups

^4^Quadratic curve analysis among B, BL, BM and BH groups

^a–c^Means within the same row without a common superscript differ significantly (*P* < 0.05)

DPI: days post infection, A: broiler chickens with basal diet and no infection, B: broiler chickens with basal diet and SE infection, BL: broiler chickens with basal diet supplemented with 300 mg/kg EOA and SE infection, BM: broiler chickens with basal diet supplemented with 500 mg/kg EOA and SE infection, BH: broiler chickens with basal diet supplemented with 800 mg/kg EOA and SE infection. EOA: coated essential oils and organic acids mixture

### Anti-*Salmonella* specific IgA and IgG concentrations

As presented in Table 8, OD value of the serum anti-SE IgG in BM group was significantly higher than that in SE-infected B group at 3 DPI (*P* < 0.05). In addition, OD value of specific IgA against *Salmonella* in the ileum digesta in BH group was significantly higher than that in BL group at 3 DPI (*P* < 0.05). Notably, no significant difference in the concentration of ileal IgA and serum IgG was observed among the five groups at 10 DPI.

**Table 8:** Effect of dietary coated essential oils and organic acids mixture (EOA) on anti-*Salmonella* specific IgG and IgA of broiler chickens (*n* = 6)

Items	Groups					SEM^1^	*P*-value		
	**A**	**B**	**BL**	**BM**	**BH**		***P1***^**2**^	**Linear**^**3**^	**Quadratic**^**4**^
Serum anti-*Salmonella* IgG (OD_450_)									
3 DPI	2.59^b^	2.62^b^	2.59^b^	2.78^a^	2.72^ab^	0.023	0.017	0.038	0.302
10 DPI	2.80	2.80	2.77	2.72	2.73	0.014	0.338	0.142	0.923
Intestinal anti-*Salmonella* IgA (OD_450_)									
3 DPI	2.76^ab^	2.77^ab^	2.68^b^	2.75^ab^	2.82^a^	0.015	0.037	0.329	0.002
10 DPI	2.75	2.80	2.78	2.71	2.75	0.014	0.286	0.138	0.875

^1^*SEM* Standard error of the mean

^2^*P1*-value represent the difference comparison between group A, B, BL, BM and BH groups

^3^Linear regression analysis among B, BL, BM and BH groups

^4^Quadratic curve analysis among B, BL, BM and BH groups

^a,b^Means within the same row without a common superscript differ significantly (*P* < 0.05)

DPI: days post infection, A: broiler chickens with basal diet and no infection, B: broiler chickens with basal diet and SE infection, BL: broiler chickens with basal diet supplemented with 300 mg/kg EOA and SE infection, BM: broiler chickens with basal diet supplemented with 500 mg/kg EOA and SE infection, BH: broiler chickens with basal diet supplemented with 800 mg/kg EOA and SE infection. EOA: coated essential oils and organic acids mixture

### Concentration of short-chain fatty acids in cecal content

As illustrated in Table 9, the concentration of isobutyric acid in the cecum digesta of negative group, BL and BH group were significantly higher than that in SE-infected group (*P* < 0.05), and adding EOA in the diet linearly increased iso-butyric acid concentration in cecal digesta of infected broilers (*P* < 0.05).

**Table 9:** Effects of dietary EOA on volative fatty acids concentration (mg/kg) in the cecal contents of broilers infected with *Salmonella* Enteritidis at 10 days post infection (*n* = 6)

Items	Groups					SEM^1^	*P*-value		
	**A**	**B**	**BL**	**BM**	**BH**		***P1***^**2**^	**Linear**^**3**^	**Quadratic**^**4**^
Acetic acid	141.45	147.19	134.28	125.09	125.35	10.136	0.954	0.503	0.978
Propionic acid	29.02	37.22	26.41	27.30	31.64	2.174	0.511	0.271	0.246
Isobutyric acid	76.49^a^	44.37^b^	83.43^a^	63.12^ab^	75.60^a^	4.567	0.040	0.026	0.164
Butyric acid	85.70	63.53	76.07	61.30	96.77	5.429	0.217	0.231	0.414
Isovaleric acid	56.25	55.78	58.87	67.78	65.29	2.430	0.432	0.144	0.893
Valeric acid	63.54	65.47	63.51	63.20	65.60	2.670	0.998	0.943	0.792

^1^*SEM* Standard error of the mean

^2^*P1*-value represent the difference comparison between group A, B, BL, BM and BH groups

^3^Linear regression analysis among B, BL, BM and BH groups

^4^Quadratic curve analysis among B, BL, BM and BH groups

^a,b^Means within the same row without a common superscript differ significantly (*P* < 0.05)

A: broiler chickens with basal diet and no infection, B: broiler chickens with basal diet and SE infection, BL: broiler chickens with basal diet supplemented with 300 mg/kg EOA and SE infection, BM: broiler chickens with basal diet supplemented with 500 mg/kg EOA and SE infection, BH: broiler chickens with basal diet supplemented with 800 mg/kg EOA and SE infection. EOA: coated essential oils and organic acids mixture

### Cecal microbiome analysis by 16S rRNA sequencing and bioinformatics

**Fig. 2:**
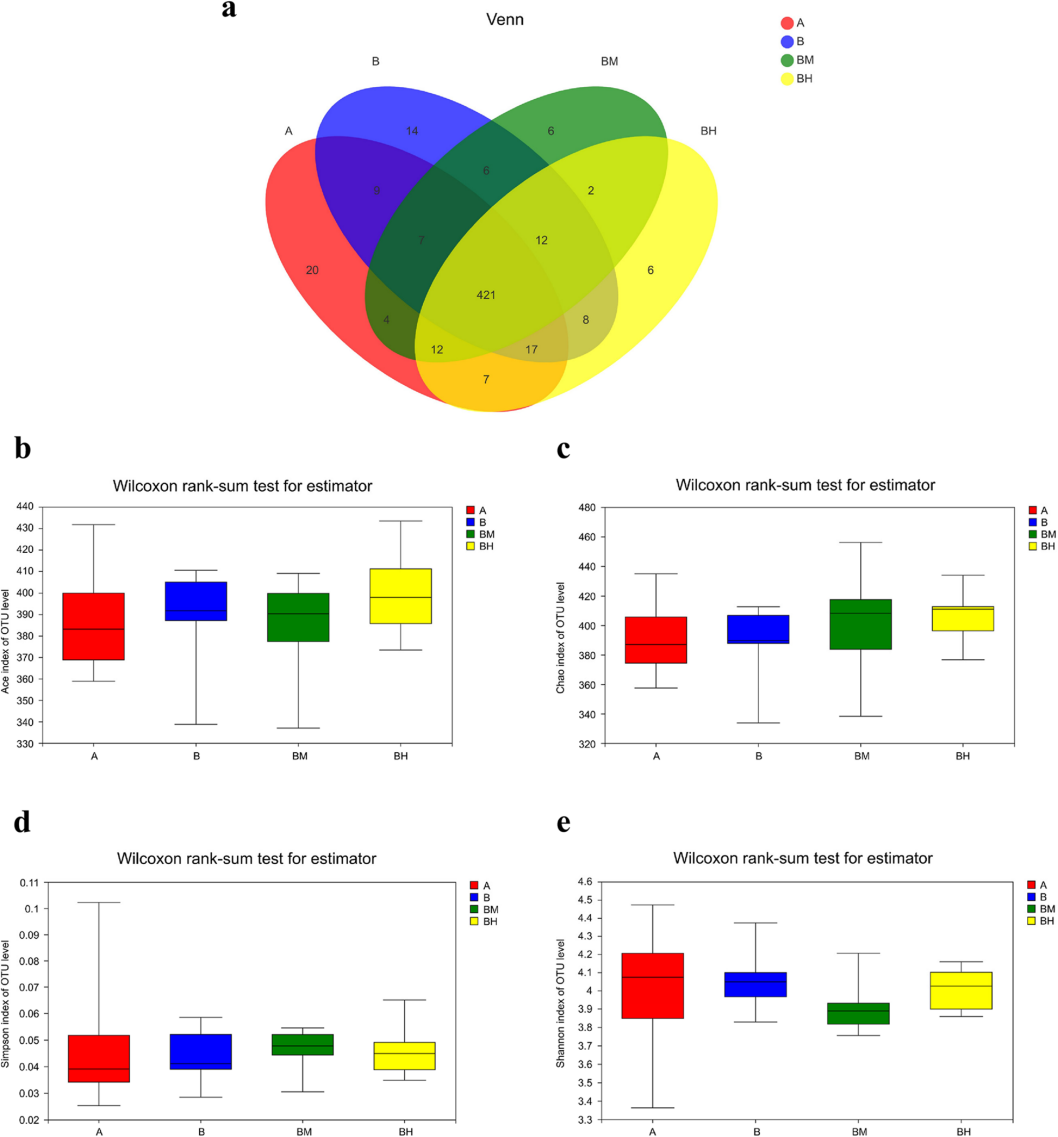
Effects of dietary supplementation EOA on the α-diversity indices of cecal microbiota communities of the SE-infected broiler chickens at 23 days of age. (**a**) Venn diagram showing the shared OTUs by groups, (**b**) Ace index, (**c**) Chao index, (**d**) Simpson index, (**e**) Shannon index. A: broiler chickens with basal diet and no infection, B: broiler chickens with basal diet and SE infection, BM: broiler chickens with basal diet supplemented with 500 mg/kg EOA and SE infection, BH: broiler chickens with basal diet supplemented with 800 mg/kg EOA and SE infection. EOA: coated essential oils and organic acids mixture. SE: *Salmonella* Enteritidis

**Fig. 3:**
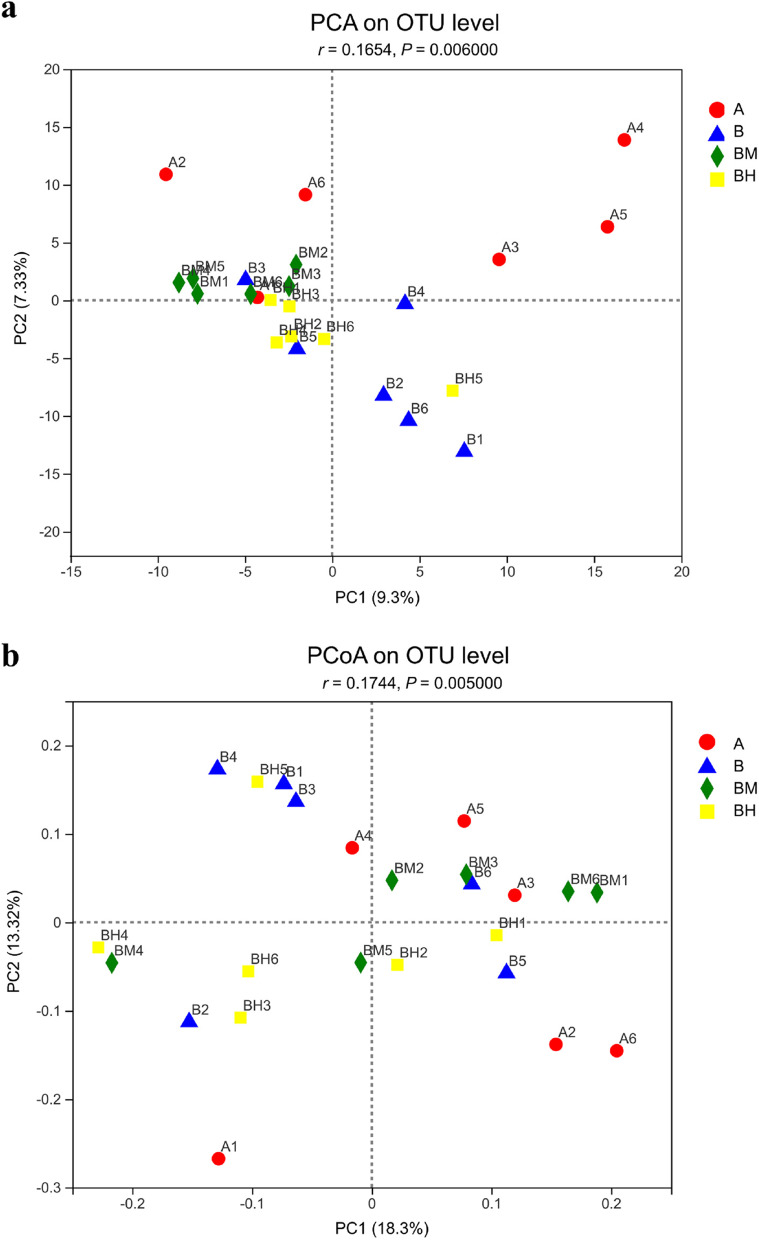
Effects of dietary supplementation EOA on the β-diversity indices of cecal microbiota communities of the SE*-*infected broiler chickens at 23 days of age. (**a**) Principal component analysis (PCA) plot of the caecal microbiota based on weighted unifrac distance, (**b**) Principal co-ordinates analysis (PCoA) plot of the caecal microbiota based on weighted unifrac distance. A: broiler chickens with basal diet and no infection, B: broiler chickens with basal diet and SE infection, BM: broiler chickens with basal diet supplemented with 500 mg/kg EOA and SE infection, BH: broiler chickens with basal diet supplemented with 800 mg/kg EOA and SE infection. EOA: coated essential oils and organic acids mixture. SE: *Salmonella* Enteritidis

In this study, 551 OTUs were obtained from ceca contents samples of the four groups based on 97% sequence similarity level. Venn diagram (Fig. 2a) indicated 421 common core OTUs were shared by the four groups, while 20, 14, 6 and 6 OTUs were unique to groups A, B, BM and BH, respectively. There were no significant differences (*P* > 0.05) in ACE index, Chao1 index, Simpson index and Shannon index among all dietary treatments (Fig. 2b–e), indicating that cecum microbial α-diversity was not influenced by EOA treatment or *Salmonella* challenge. In order to study the similarity or difference of cecum microbial community structure in different samples, the β-diversity of cecal microorganisms was assessed by PCA analysis and PCoA analysis. PCA analysis showed that there was significant separation in cecal microbial community structure among the four groups (*P* = 0.006) (Fig. 3a and b), especially between the infected control and non-infected control, and between the infected control and the BM group.

**Fig. 4:**
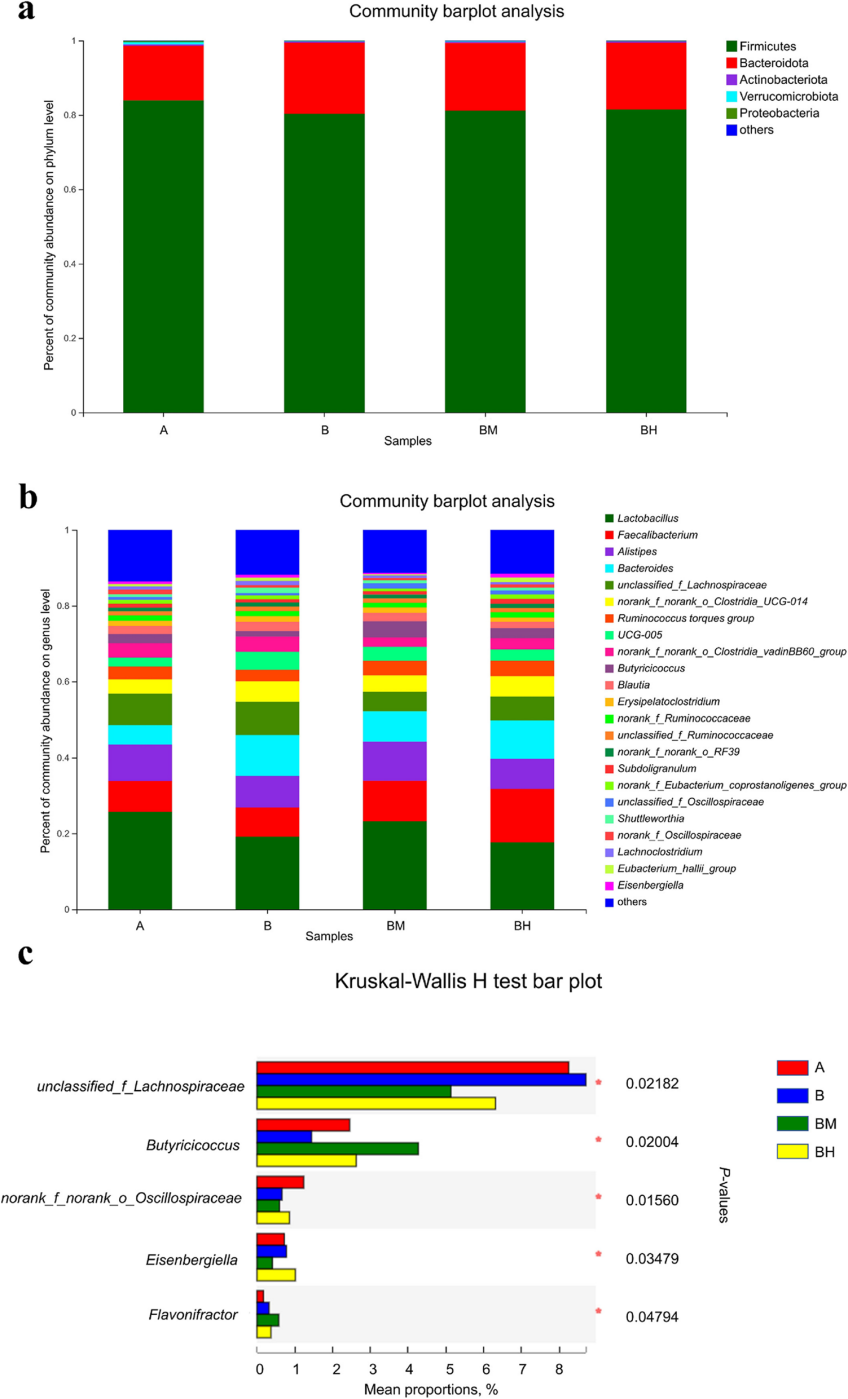
Relative abundance of cecal microbial composition of broiler chickens from different treatment groups. (**a**) Composition of caecal microbiota of the broilers at the phylum level, (**b**) Composition of caecal microbiota of the broilers at the genus level, (**c**) Differential cecal microbiota at different taxa levels among different groups. Asterisks (^∗^*P* < 0.05, ^∗^^∗^*P* < 0.01) indicate statistical differences between the treatment group. A: broiler chickens with basal diet and no infection, B: broiler chickens with basal diet and SE infection, BM: broiler chickens with basal diet supplemented with 500 mg/kg EOA and SE infection, BH: broiler chickens with basal diet supplemented with 800 mg/kg EOA and SE infection. EOA: coated essential oils and organic acids mixture. SE *Salmonella* Enteritidis

As presented in Fig. 4a, at the phyla level, ceca microbiota was dominated by Firmicutes (81.72%), Bacteroidota (17.50%), Actinobacteriota (0.34%), followed by Verrucomicrobiota (0.23%) and Proteobacteria (0.16%) for all treatments, with no significant differences in the relative abundance among four treatment groups (*P* > 0.05). At the genus taxa, the top 10 genera in abundance were *Lactobacillus* (21.44%), *Faecalibacterium* (10.17%), *Alistipes* (9.05%), *Bacteroides* (8.44%), *unclassified_f_Lachnospiraceae* (7.10%), *norank_f_norank_o_Clostridia_UCG-014* (4.72%), *Ruminococcus torques group* (3.59%), *UCG-005* (3.42%), followed by *norank_f_norank_o_Clostridia_vadinBB60_group* (3.32%) and *Butyricicoccus* (2.70%) (Fig. 4b). The comparison of cecal bacterial compositions among four groups showed that the relative abundance of *unclassified_f_Lachnospiraceae* was significantly (*P* < 0.05) increased in the single SE-infected group, while the relative abundance of *Butyricicoccus* was significantly (*P* < 0.05) increased in BM group. In addition, the relative abundances of *norank_f_Oscillospiraceae*, *Eisenbergiella* and *Flavonifractor* were significantly (*P* < 0.05) increased in the non-infected group, BH group and BM group respectively (Fig. 4c). *Salmonella* infection also significantly (*P* < 0.05) decreased the relative abundance of *norank_f_norank_o_Oscillospiraceae, norank_f_norank_o_Rhodospirillales* and *Eggerthella*. However, dietary EOA treatment significantly (*P* < 0.05) increased relative abundance of *Butyricicoccus*, *unclassified_f_Oscillospiraceae*, *Anaerotruncus*, *unclassified_f_Bacillaceae* and *Enterococcus*, whereas decreased relative abundance of *unclassified_f_Lachnospiraceae*, *norank_f_norank_o_Clostridia_vadinBB60_group*, *Eisenbergiella*, *UCG-009* and *Merdibacter* (*P* < 0.05).

**Fig. 5:**
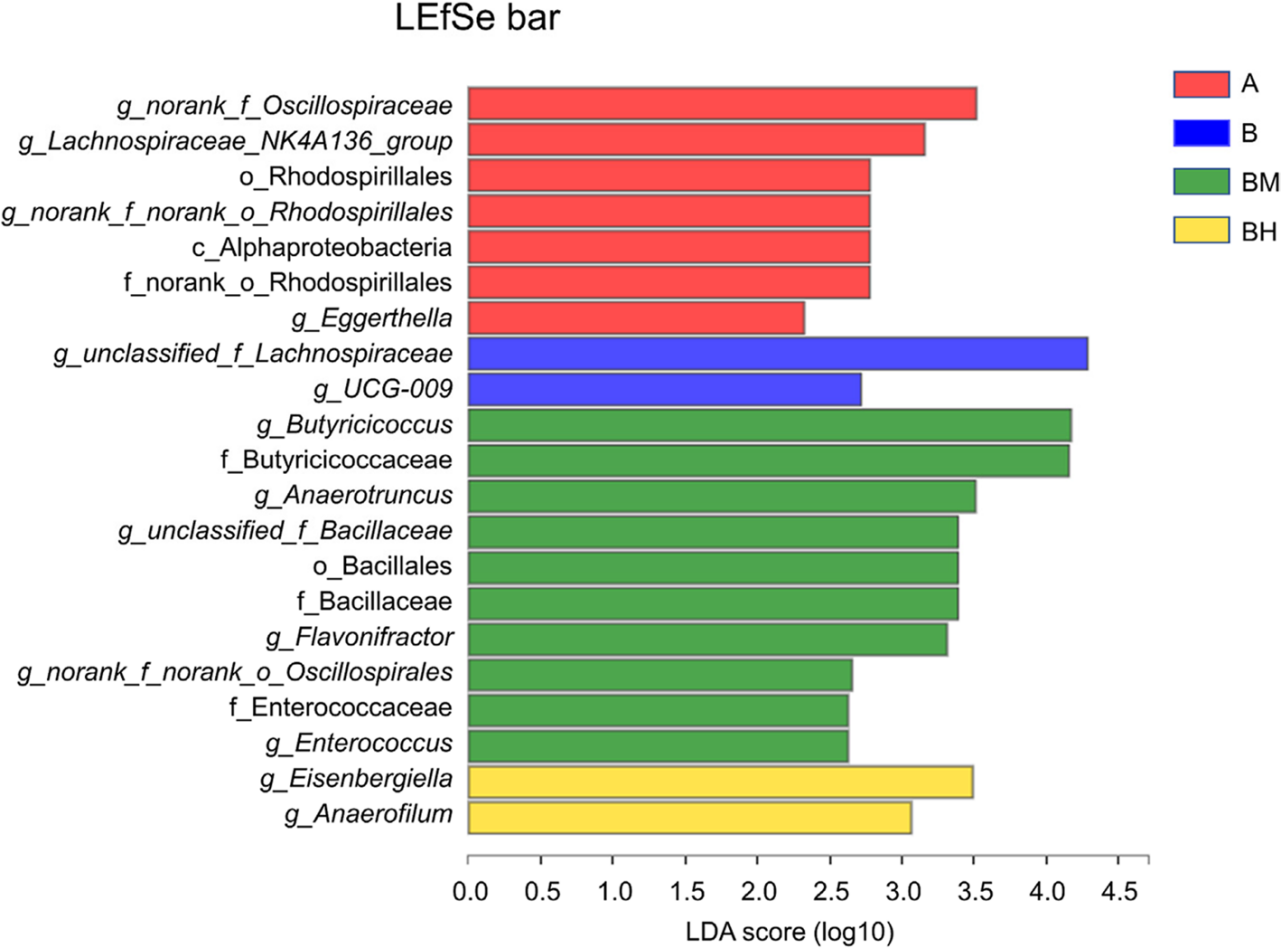
Histogram of the Linear Discriminant Analysis (LDA) score computed for differentially abundant taxa with cut-off LDA score > 2.0. The different colors represent microbial groups that play a significant role in groups A, B, BM and BH. A: broiler chickens with basal diet and no infection, B: broiler chickens with basal diet and SE infection, BM: broiler chickens with basal diet supplemented with 500 mg/kg EOA and SE infection, BH: broiler chickens with basal diet supplemented with 800 mg/kg EOA and SE infection. EOA: coated essential oils and organic acids mixture. SE: *Salmonella* Enteritidis

LEfSe analysis (Fig. 5) showed that *g_norank_f_Oscillospiraceae, g_Lachnospiraceae_NK4A136_group, g_Eggerthella,* f_norank_o_Rhodospirillales*, g_norank_f_norank_o_Rhodospirillales,* o_Rhodospirillales and c_Alphaproteobacteria were significantly (*P* < 0.05) enriched in the non-infected group, while *g_unclassified_f_Lachnospiraceae* and *g_UCG-009* were significantly (*P* < 0.05) enriched in the positive B group. Moreover, *g_Butyricicoccus, *f_Butyricicoccaceae, *g_Anaerotruncus, g_norank_f_norank_o_Oscillospirales, g_unclassified_f_Bacillaceae, *o_Bacillales*, *f_Bacillaceae, *g_Flavonifractor, *f_Enterococcaceae and *g_Enterococcus* were significantly (*P* < 0.05) enriched in the BM group, and *g_Eisenbergiella* and *g_Anaerofilum* were significantly (*P* < 0.05) enriched in the BH group.

**Fig. 6:**
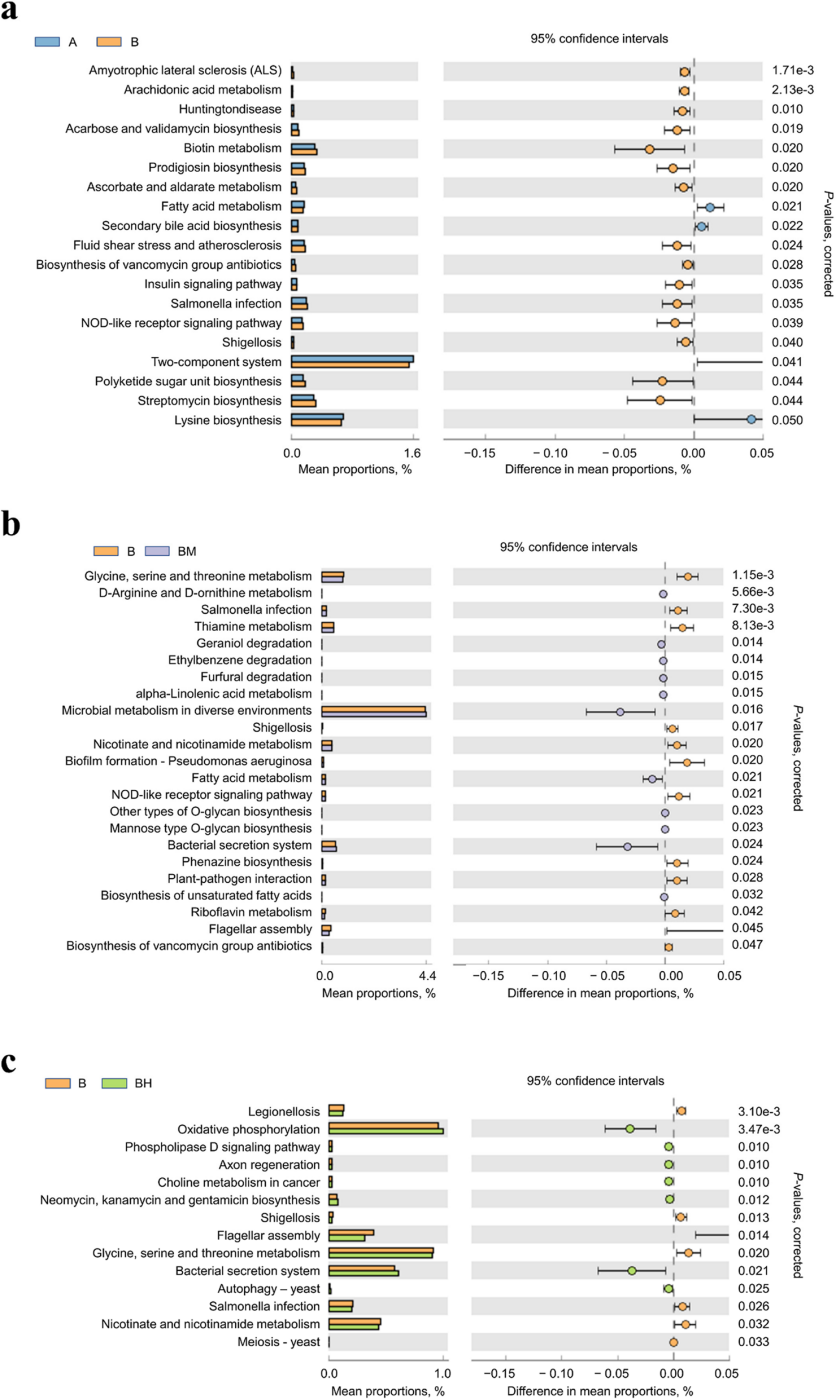
PICRUSt metagenome inference analysis based on 16S rRNA dataset: (**a**) A vs. B, (**b**) B vs. BM, and (**c**) B vs. BH. (**a**–**c**) Prediction of significant KEGG pathways (level 3) that were differentially regulated in SE-infected group compared to non-infected group (*P* < 0.05). Mean proportion of functional pathways is illustrated with bar plots and dot plots indicate the differences in mean proportions between two groups based on *P*-values obtained from two-sided Welch’s *t*-test. A: broiler chickens with basal diet and no infection, B: broiler chickens with basal diet and SE infection, BM: broiler chickens with basal diet supplemented with 500 mg/kg EOA and SE infection, BH: broiler chickens with basal diet supplemented with 800 mg/kg EOA and SE infection. EOA: coated essential oils and organic acids mixture, KEGG: Kyoto encyclopedia of genes and genomes, SE: *Salmonella* Enteritidis

PICRUSt analysis exhibited that functions related to microbial infection and anti-infection such as *Salmonella* infection, *Shigellosis*, nucleotide oligomerization domain-like (NOD-like) receptor signaling pathway, streptomycin biosynthesis, prodigiosin biosynthesis, acarbose and validamycin biosynthesis, biotin metabolism, ascorbate and aldarate metabolism, biosynthesis of vancomycin group antibiotics and insulin signaling pathway, were significantly enhanced in single SE-infected B group compared with the non-infected A group (*P* < 0.05) (Fig. 6a). Comparing with the single SE-infected B group, *D*-arginine and *D*-ornithine metabolism, ethylbenzene degradation, furfural degradation, alpha-linolenic acid metabolism, microbial metabolism in diverse environments, fatty acid metabolism, bacterial secretion system and biosynthesis of unsaturated fatty acids were significantly enhanced in EOA-treated group (*P* < 0.05), while *Salmonella* infection, thiamine metabolism, Shigellosis, NOD-like receptor signaling pathway, flagellar assembly and biosynthesis of vancomycin group antibiotics were significantly enriched in single SE-infected B group (*P* < 0.05) (Fig. 6b and c).

**Fig. 7:**
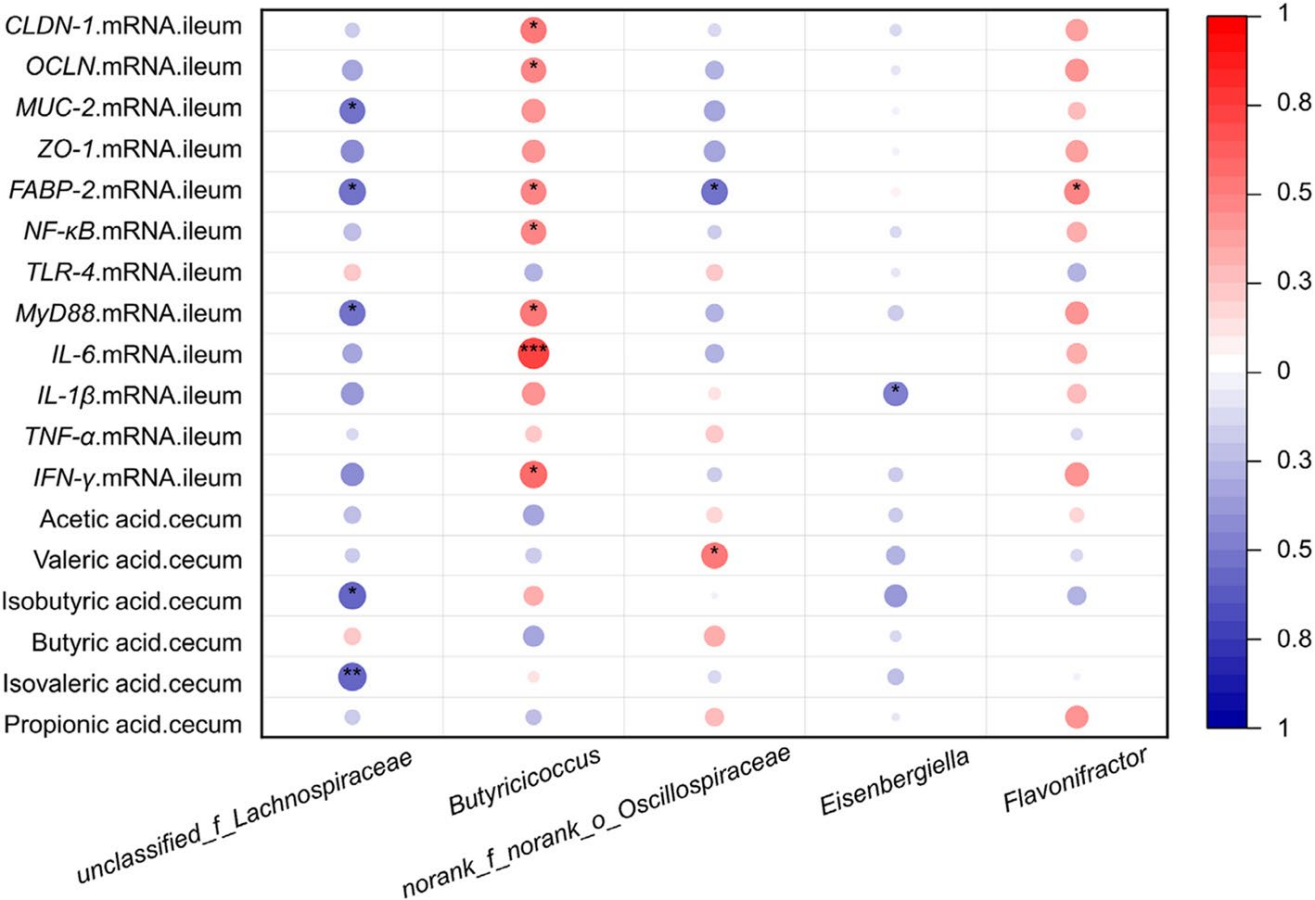
Spearman’s correlation analysis between phenotypic variables and the relative abundance of microbial communities with significant differences (genus level, *n* = 6/group). The color and dot size represent the correlation coefficient within rows. ^*^P < 0.05, ^**^P < 0.01

It is vital to construct a network between the differential microbiota and the expressions of intestinal tight junction protein genes and immune-related genes together with SCFA concentration of cecal content to understand how the intestinal host-microbial relationship regulates host defense and inflammation (Fig. 7). Results of the Spearman’s correlation coefficients showed that the relative abundances of *unclassified_f_Lachnospiraceae* (significantly enriched in *Salmonella*-infected chickens) was markedly negatively correlated with the relative mRNA expression levels of *MUC-2*, *FABP-2* and *MyD88*, and concentration of isobutyric acid and isovaleric acid in cecal content (*P* < 0.05 or *P* < 0.01). The *Butyricicoccus* showed a positive regulatory effect on the mRNA expression of *CLDN-1*, *OCLN*, *FABP-2*, *NF-κB*, *MyD88*, *IL-6* and *IFN-γ* (*P* < 0.05 or *P* < 0.01), while the relative abundance of *g_norank_f_Oscillospiraceae* had a negative correlation with the relative mRNA expression of *FABP-2*, but displayed a positive correlation with the concentration of valeric acid. In addition, the significant positive correlation between the relative abundances of *g_Flavonifractor* and the relative mRNA expression of *FABP-2* was observed (*P* < 0.05).

## Discussion

Since chickens serve as the reservoir of *Salmonella*, innovative on-farm non-antibiotic strategies for reducing pathogen colonization in birds are critical for reducing the contamination of poultry meat and eggs together with controlling human infections. Essential oils and short-chain fatty acids, used as potential antibiotics alternatives have received great attentions, in view of their potential antimicrobial properties and anti-inflammatory potential in broilers [40–42]. Herein, the present study assessed the efficacy of a new blend of coated essential oils and organic acids on growth performance, colonization and invasion of *Salmonella* as well as intestinal health of broilers infected with SE, and then the action mechanism was further explored.

Our results revealed that single *Salmonella* infection notably increased the feed to gain ratio during the later and the whole phase. Similar observations were obtained in some previous studies [43, 44]. Increased FCR induced by *Salmonella* challenge in our study might be due to a numerical reduce in ABW. Meanwhile, our study also indicated that dietary supplementation with 800 mg/kg of EOA remarkably improved feed efficiency of SE-infected chickens through numerically decreasing feed intake but without obvious effect on ABW during the later and the whole phase compared with the infected control birds, indicating that supplemental EOA could alleviate the negative effects caused by SE infection. Similarly, several studies have also reported an improvement in the body weight, and/or feed conversion rate in non-challenged broilers after feeding different EOA products. For example, Liu et al. [45] reported that dietary supplementation with protected essential oils and organic acids mixture containing citric acid, malic acid, sorbic acid, fumaric acid, thymol, vanillin and eugenol significantly improve FCR due to reducing average daily feed intake, but had no effects on ADG. Abdelli et al. [46] also showed that microencapsulated fumaric acid, thymol, or their mixture improved the overall FCR. Inversely, other studies suggested that dietary essential oils and organic acid blend inclusion had no obvious effects on growth performance in broiler chickens challenged with pathogens or not. For instance, a specific blend of EO based on a mixture of cinnamaldehyde and thymol alone or in combination with sodium butyrate did not affect growth performance of broiler chickens infected with SE, but significantly reduced *Salmonella* colonization in the cecum [25]. Adewole et al. [47] observed that dietary EOA treatments had no effect on ADFI and FCR at all phases in broiler chickens. Inconsistent results in growth performance across studies might be attributable to several factors, including the nature of essential oils and organic acids, chemical composition and dosage of EOA mixture used, protected EOA or unprotected, diet composition and digestibility, age and genetic background of the bird, health status, as well as characteristics of infection pathogen and challenge route. However, our findings suggested that the EOA supplementation could be effective in minimizing the negative impact on growth performance and FCR due to *Salmonella* infection.

Intestinal morphology, intestinal potential pathogens load and intestinal bacterial translocation together with intestinal immune responses are important indicators for assessing intestinal health, barrier integrity and functionality, and also be involved in the function of intestinal digestion and absorption [28, 48]. In this study, SE infection damaged the morphology of ileal villi, significantly reduced VH/CD ratio and promoted the growth of intestinal potential pathogens such as *Salmonella, Escherichia coli,* and *Campylobacter* at 3 and 10 DPI, which was in agreement with previous findings [49, 50]. Meanwhile, SE infection also induced intestinal inflammation by upregulating inflammatory-related cytokine *TNF-α* mRNA levels, pro-inflammatory cytokines *IL-1β* and *NF-κB* mRNA levels in the ileum at the early infection stage. Furthermore, SE infection also led to severe intestinal barrier function injury, as indicated by downregulated the expressions of intestinal tight junction proteins genes, such as *CLDN-1*, *OCLN*, *ZO-1* together with *MUC-2* obtained in our study. Totally, our observations indicated that SE infection caused intestinal inflammation and barrier dysfunction, resulting in damaged intestinal health in broiler chickens, which was in agreement with previous findings from chickens’ studies [51–55]. Nevertheless, SE-induced changes in the gut observed in the current study were reversed or mitigated by EOA administration, as evidenced by improved villus height and VH/CD in the ileum, and reduced *Salmonella* load in the cecum and internal organs. Meanwhile, dietary EOA treatment also upregulated *CLDN-1*, *OCLN*, *ZO-1*, *MUC2* and *FABP2* mRNA levels at the middle infection phase, as well as linearly reduced the gene expression level of *TLR4*, *NF-κB* and *IL-1β* at the early infection stage in infected broiler chickens. In accordance with our findings, plenty of studies have demonstrated that in-feed protected essential oils and organic acids blend administration could alleviate *Salmonella*-induced harmful effects on intestinal health through suppressing intestinal potential pathogen colonization and invasion [25, 26, 56, 57], reducing intestinal inflammatory responses, improving intestinal morphological structure [26, 28, 58, 59], and upregulating tight junction proteins genes expression [13, 60–62]. Additionally, increased amount of ileal anti-SE IgA and serum anti-SE IgG was also observed in the SE-infected chickens fed the medium and higher dose of EOA in our study. Nevertheless, different from the result of Zhang et al. [26], reported that dietary EOA administration had no difference in IgA content of the jejunum of SE-infected special pathogen-free birds. Several reports have indicated that cell mediated immunity is responsible for the clearance of *S.* Enteritidis from the tissue [63, 64], while humoral immunity such as intestinal IgA is critical for the limitation of intestinal pathogens such as *Salmonella* colonization, serum IgG directly contributes to an immune response including neutralization of toxins of pathogens [65, 66]. Based on above obtained findings, our data indicated that the EOA reduce *Salmonella* colonization and invasion in the gut, possibly related to more production of IgA in the gut of broiler chickens. Moreover, our results also suggested that dietary EOA addition improved FCR, possibly due to mitigating gut inflammation and gut injury caused by SE infection.

Surprisingly, in our study, SE infection enriched the relative level of *Lactobacillus* in the cecum compared with the non-infected control birds, which was similar to previous observation from Videnska [67]. Videnska et al. reported that SE infection increased cecal Lactobacillaceae relative abundance, but conflicted with other reports, which found the reduced beneficial bacteria such as *Lactobacillus*, *Bifidobacterium* numbers in the cecal contents following *Salmonella* infection [3, 49, 68, 69]. This pattern of changes would indicate serious dysbiosis in the composition of the intestinal microflora in *Salmonella*-infected chickens. The increase of *Lactobacillus* in the single SE-infected birds could be attributable to the microaerophilic growth of *Lactobacilli*, which may allow them to survive under conditions of increased redox potential due to the production of reactive oxygen species by granulocytes infiltrating the site of inflammation as occurs in a highly inflamed gut [67, 70]. Inversely, infected chickens fed diets supplemented with different concentrations of EOA exhibited a decrease in *Lactobacillus* counts in cecal digesta at whether 3 DPI or 10 DPI. Our data indicated that EOA could balance the intestinal ecosystem and reduce the dysbiosis, resulting in restoration of ecological balance of intestinal microflora. Likewise, a study also found that butyrate supplementation reduced intestinal *Lactobacillus* concentration in *Salmonella*-infected chickens [22, 71]. *Lactobacillus* spp. are one of the most abundant commensal bacteria in the gut. Decreased population of certain *Lactobacillus* spp. carrying gene encoding bile salt hydrolase in the cecal contents might explain the reason why the inclusion of EOA improved FCR of SE-infected chickens. Overall, a notable reduction in gut *Salmonella* load, along with gut morphological impairment induced by *Salmonella.* A remarkable increase in ileal specific IgA and intestinal TJ expression levels obtained from the *Salmonella*-infected chickens fed EOA, showing that the inclusion of the product EOA not only could alleviate SE-induced intestinal injury, but also is effective in providing protection against SE infection in broiler chickens. The findings also indirectly contribute to food safety together with reducing incidence of horizontal transmission of *Salmonella* infection. These observations obtained in our study may be directly associated with the antimicrobial and anti-inflammatory activity of EOs [72] or OAs in the gastrointestinal tract of chickens [41, 73–75] as well as downregulating *Salmonella* virulence genes expression capacity of EOs or OAs [41, 42] in the EOA product.

Numerous studies have confirmed that gut microbiota and their metabolites directly or indirectly by influence host’s immune and health [76, 77]. Additionally, many studies have showed that modifying the microflora balance in the gastrointestinal tract through nutritional strategies may improve gut barrier function and enhance bird’s resistance to colonization by *Salmonella* and other pathogens [28, 51]. In this study, SE infection alone remarkably reduced the concentration of isobutyric acids in the cecal digesta, whereas dietary supplementation of EOA tend to linearly increase isobutyric acid content in cecal digesta of broilers relative to the infected treatments, which was in agreement with previous study [78]. Moreover, the addition of high dose EOA also increased butyric acid content to some extent. Intestinal commensal microbes and SCFA, especially butyrate acid was reported to inhibit *Salmonella* colonization in the ceca [79] and downregulate the invasion and virulence genes expression of *Salmonella* in chickens [41, 42, 80]. Thus, our data suggested that EOA addition contribute to beneficial effects on gut health, possibly due to increasing the contents of isobutyric acid and butyric acid in the cecum of the *Salmonella*-infected broilers.

In the current study, neither SE infection nor EOA treatment altered α-diversity, while PCA analysis showed that SE infection obviously changed cecal microbial β-diversity relative to the negative non-infected control, indicating that SE infection notably disturbed microbial community structure of gut microbiota of chickens. In addition, interestingly, taxa analysis found that relative to the uninfected control, SE infection alone significantly expanded relative abundance of *unclassified_f_Lachnospiraceae*, which was similar to our observation from bacterial culture, whereas decreased the relative abundance of *norank_f_norank_o_Oscillospiraceae, norank_f_norank_o_Rhodospirillales* and *Eggerthella*. Although members of *Lachnospiraceae* are one of the major producers of short-chain fatty acids, different groups are positively correlated with gut health [81], extraintestinal diseases [82] and metabolic disorders [83]. *Oscillospiraceae* is a key bacterium in the pathogenesis of rheumatoid arthritis, which was be negatively associated with gut health [84]. Over-presentation of butyrate-producing *Lachnospiraceae* in the cecum showed that SE infection stimulates the immune system, allowing the proliferation of *Lachnospiraceae* as a biofilm to defend against pathogen infection and further confirming our observations from bacterial culture. Therefore, our data showed that SE infection altered the composition of cecal microbiome, resulting in inducing intestinal dysbiosis and intestinal inflammation, which was in similar with previous observations from chickens [3, 5, 49, 67]. While the medium-dose of EOA also notably altered cecal microbial β-diversity as compared with the infected control, which was similar to previous result [85], but was inconsistent with other previous observations [27, 58], possibly due to the differences in EOA formula or challenged model. Meanwhile, the inclusion of appropriate dose of EOA could alter cecal microbial community structure of the infected chickens. Taxa and LEfSe analysis found that dietary supplementation with suitable EOA significantly increased relative abundance of Firmicutes*, g_Butyricicoccus*, *g_Anaerotruncus, g_unclassified_f_Bacillaceae, g_Enterococcus*, whereas decreased relative abundance of Bacteroidetes, *unclassified_f_Lachnospiraceae*, *norank_f_norank_o_Clostridia_vadinBB60_group*, *Eisenbergiella*, *UCG-009* and *Merdibacter*. Members of Bacteroidetes mainly produce acetic acid and propionic acid through hydrolysing a variety of indigestible dietary carbohydrates such as non-starch polysaccharides and resistant starch [86], which was associated with gut health and metabolism, while Firmicutes mainly produce butyric acid and was positively correlated with obesity, good FCR and gut health [28, 62]. *Butyricicoccus* is a Gram-positive, strictly anaerobic *Clostridium* cluster IV bacterium that produces high levels of butyrate and resists *Salmonella* infection, and its abundance is positively correlated with intestinal health [87]. Butyric acid-producing bacteria *g_Anaerotruncus,* is usually positively associated with obesity [88]. Some *Enterococcus* strains are normal resident commensal bacteria in the intestinal tract of food animals and human, which were positively associated with gut health and usually used as antibiotics alternatives in animal and poultry production, while other *Enterococcus strains* could invade into internal organs to cause malignant infection in humans and animals, especially when antibiotics are overused [89–91]. *Eisenbergiella* is a degrader of complex polysaccharides and producer of SCFA, which are involved in gut health and bile acid metabolism [92]. Thus, higher proportion of Firmicutes, *Butyricicoccus*, *Anaerotruncus*, and *Enterococcus*, accompanied by lower relative abundance of Bacteroidetes, *unclassified_Lachnospiraceae, Eisenbergiella* in the cecum of *Salmonella*-infected broiler chickens following EOA administration, suggesting that pretreatment with EOA control *Salmonella* infection and improve feed efficiency, possibly via improving gut microbiome and increasing the abundance of SCFA-producing bacteria. These results further confirmed our above observations. These data also implied that the health-improving effects of EOA on *Salmonella*-infected broiler chickens might be positively associated with the restoration of intestinal microbiota balance.

PICRUSt analysis indicated that compared with the non-infected group, SE infection increased abundances of cecal microbial functions involved in microbial infection and anti-infection such as *Salmonella* infection, *Shigellosis*, NOD-like receptor signaling pathway, flagella assembly, streptomycin biosynthesis, prodigiosin biosynthesis, acarbose and validamycin biosynthesis, biotin metabolism, ascorbate and aldarate metabolism, biosynthesis of vancomycin group antibiotics and insulin signaling pathway. NOD-like receptor signal pathway was involved in innate immune response, inflammation and apoptosis [93]. Ascorbate and aldarate metabolism were involved in antioxidant function. Hence, we speculated that *Salmonella* infection induced intestinal inflammatory responses and oxidative stress, increased metabolism of some nutrients and stimulated antibiotics biosynthesis in broiler chickens, all of these changes possibly contribute to reasonable reasons why *Salmonella* infection usually decreased feed efficiency of broiler chickens, caused gut damage and increased the occurrence of antibiotics-resistant bacteria. Fatty acid metabolism and biosynthesis of unsaturated fatty acids was involved in anti-oxidative, anti-inflammatory and anti-infective activities [94, 95]. These changes in functions of gut microbiota in the SE-infected chickens after feeding moderate dose of EOA group indicated that the coated essential oils and organic acid additives possesses anti-inflammatory and anti-infective capacities through modulating the functions of gut microbiota. Meanwhile, the enriched pathways related to neomycin, kanamycin and gentamicin biosynthesis in the high dose of EOA group, indicated that supplemental high level of EOA could effectively mobilize the bactericidal mechanism of gut microbiota, resulting in promoting the production of bacteriostatic substances, which may be one of the reasons for the effective reduction of intestinal *Salmonella* infection by adding EOA. Spearman’s correlation analysis found that the relative mRNA expression levels of *MUC-2*, *FABP-2* and *MyD88*, together with concentration of isobutyric acid and isovaleric acid had a negative relationship with *unclassified_f_Lachnospiraceae*, while the relative mRNA expression of *CLDN-1*, *OCLN*, *FABP-2*, *NF-κB*, *MyD88*, *IL-6* and *IFN-γ* had a positive relationship with *Butyricicoccus*. Some reports have showed that *unclassified_f_Lachnospiraceae* was associated with the destruction of tight junctions and aggravation of inflammation [96], whereas *Butyricicoccus* was positively related to the enhancement of epithelial barrier function and relief of colitis in rats [97]. Thus, we suggested that the health-improving effects of EOA on *Salmonella*-infected broiler chickens might attribute to increasing intestinal *Butyricicoccus* relative abundance. Further research is necessary to confirm our hypothesis.

## Conclusions

In summary, dietary supplementation with coated essential oils and organic acids mixture at 500 mg/kg and 800 mg/kg could alleviate the harmful effects caused by SE infection through improving intestinal morphology; reducing *Salmonella* load in liver and spleen and cecum; up-regulating ileal *CLDN-1*, *OCLN*, *ZO-1*, *MUC-2* and *FABP-2* mRNA levels whereas down-regulating *TLR-4* and *TNF-α* mRNA levels; increasing cecal isobutyric acid concentration and the relative abundance of *Butyricicoccus* and *Anaerotruncus* in the cecum; along with enriching alpha-linolenic acid metabolism, fatty acid metabolism and biosynthesis of unsaturated fatty acids of gut microbiota. Overall, the inclusion of the compound preparation of coated essential oil and organic acids in the diets might be an effective strategy to alleviate the negative effects caused by SE infection.

## Data Availability

The 16S rRNA gene sequencing data generated and analyzed during the current study are available in the NCBI primary data archive (PDA) with accession number PRJNA 915,671. This data can be found here: https://www.ncbi.nlm.nih.gov/bioproject/915671.

## Abbreviations

AA: Arbor Acres
ABW: Average body weight
ADFI: Average daily feed intake
ADG: Average daily gain
CD: Crypt depth
CFU: Colony-forming unit
CLDN-1: Claudin-1
DPI: Days post infection
ELISA: Enzyme-linked immune sorbent assay
EOs: Essential oils
EOA: Coated essential oils and organic acids mixtures
FABP-2: Fatty acid binding protein
FCR: Feed conversion rate
HRP: Horseradish peroxidase
IFN-γ: Interferon-γ
IgA: Immunoglobulin A
IgG: Immunoglobulin G
IL-6: Interleukin-6
IL-1β: Interleukin-1β
LDA: Linear discriminant analysis
LEfSe: Linear discriminant analysis combined effect size
MOT: Mortality
MUC-2: Mucin-2
MyD88: Myeloid differential protein-88
NF-κB: Nuclear factor kappa-light-chain-enhancer of activated B cells
NOD: Nucleotide oligomerization domain
NRC: National research council
OAs: Organic acids
OCLN: Occludin
OD: Optical density
OTUs: Operational taxonomic units
PBS: Phosphate buffered saline
PCA: Principal components analysis
PCoA: Principal coordinate analysis
PICRUSt: Phylogenetic investigation of communities by reconstruction of unobserved states
QIIME: Quantitative insights into microbial ecology
RT-PCR: Reverse transcription-rolymerase chain reaction
SCFA: Short-chain fatty acids
SEM: Standard error of the mean
SE: *Salmonella* Enteritidis
SPF: Special pathogen-free
TLR-4: Toll-like receptor-4
TNF-α: Tumor necrosis factor-α
VH: Villus height
VH/CD: Villus height to crypt depth ratio
ZO-1: Zonula occludens-1
